# Supplementary material for: Molecular Hydrogen Enhances Proliferation of Cancer Cells That Exhibit Potent Mitochondrial Unfolded Protein Response
Source: Int J Mol Sci. 2022 Mar 7;23(5):2888. doi: 10.3390/ijms23052888 (PMC8910898; doi:10.3390/ijms23052888)
Supplement: Supplementary file 1 [file ijms-23-02888-s001.zip › ijms-1619681-supplementary.pdf]

Supplementary Materials

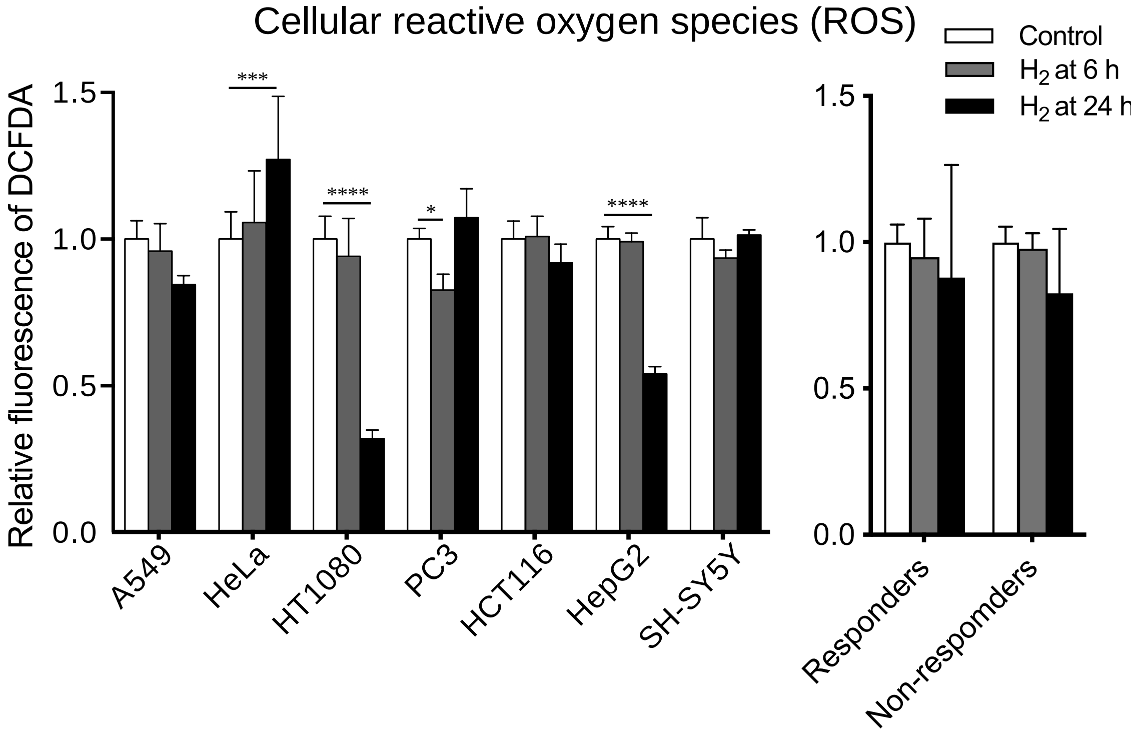

Figure S1. Change of cellular ROS levels by hydrogen gas. Relative fluorescence of DCFDA was measured using flowcytometry at 6 and 24 h under 1% hydrogen gas. Mean and SD are indicated (n = 3 culture dishes). *$p < 0.05$, **$p < 0.01$, ***$p < 0.001$ and ****$p < 0.0001$ by two-way repeated measures ANOVA followed by Dunnett's multiple comparison test compared to control.

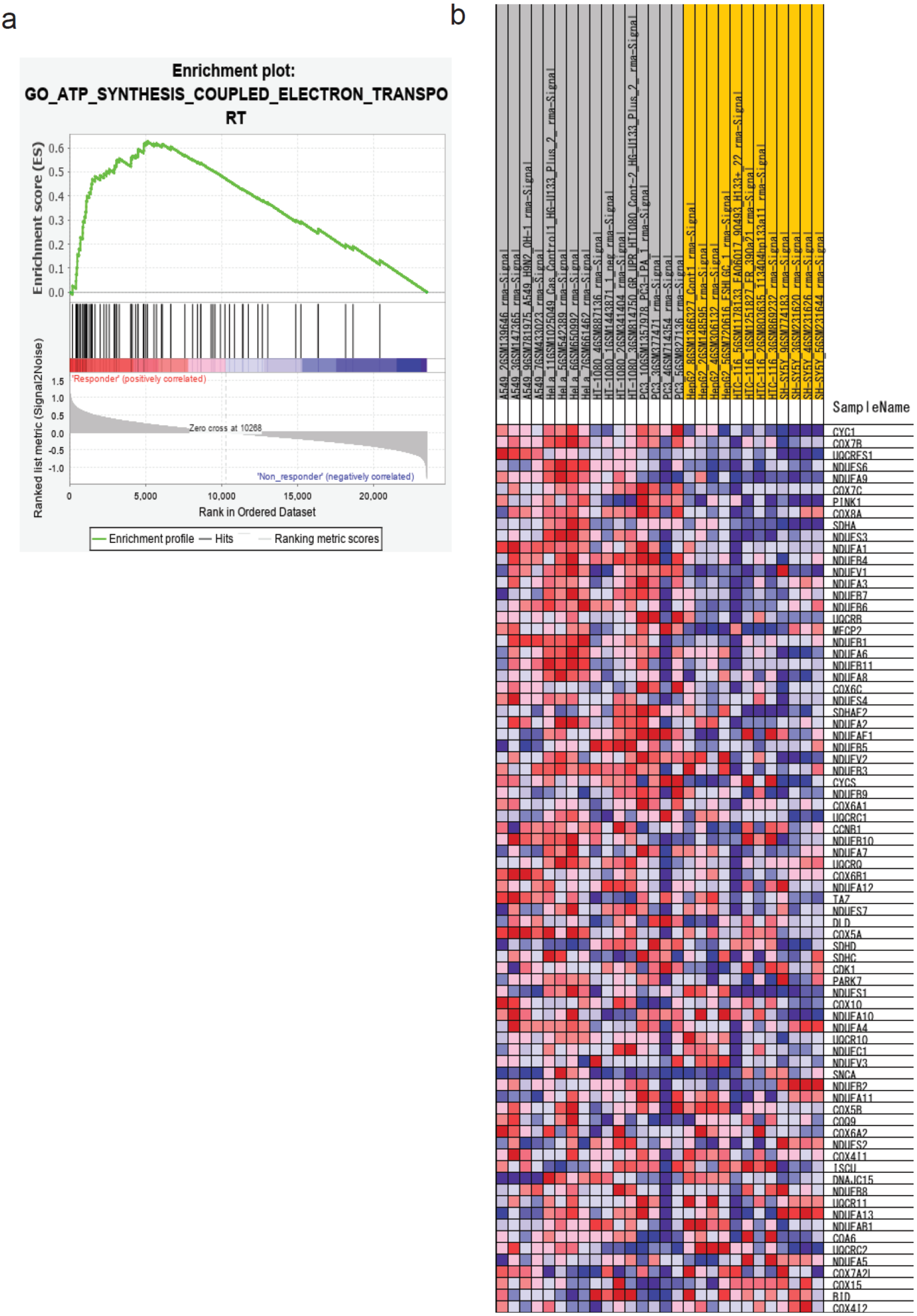

Figure S2. Gene sets enriched in the responders. An enrichment plot (**a**) and a heatmap (**b**) of "ATP synthesis coupled electron transport" that was the most enriched gene set in the responders (see Figure 3a).

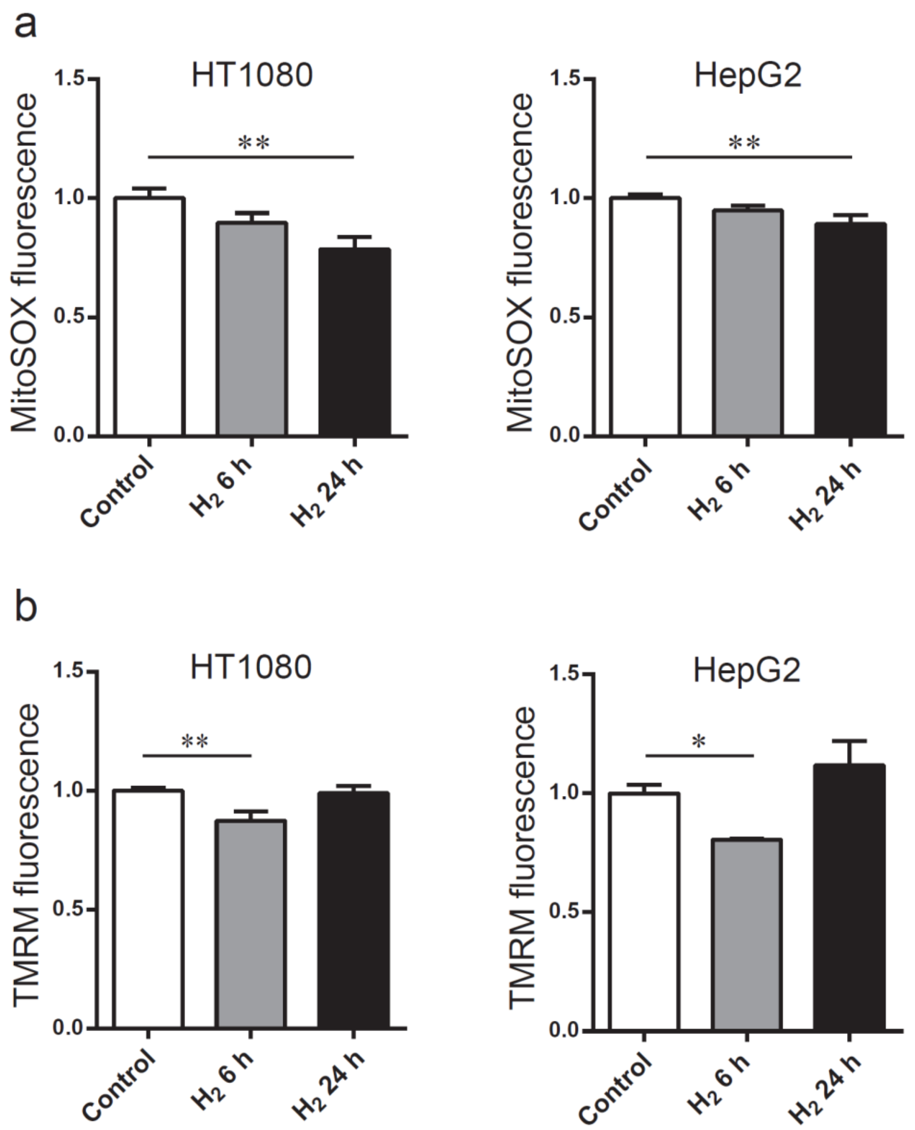

Figure S3. Mitochondrial superoxide (MitoSOX) and mitochondrial membrane potential (TMRM) in HT1080 cells (a responder) and HepG2 cells (a non-responder) under 10% hydrogen gas. Relative fluorescence of MitoSOX (**a**) and TMRM (**b**) was measured using flowcytometry after 6- and 24-h incubation under 10% hydrogen gas. Mean and SD are indicated (n = 3 culture dishes). *$p < 0.05$ and **$p < 0.01$, by one-way ANOVA followed by Dunnett's multiple comparison test compared to control.

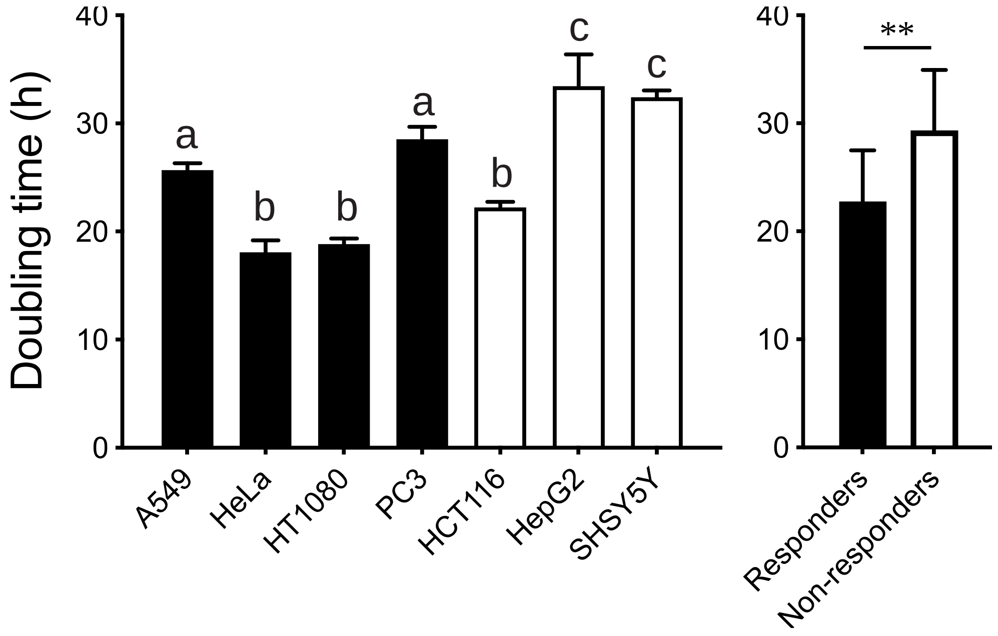

Figure S4. Doubling time of seven cell lines. Mean and SD are indicated (*n* = 3 culture dishes). Single letter labels indicate $p < 0.05$ by one-way ANOVA followed by Tukey's multiple comparison test. Values in the same label (for example, two "a's") are not statistically different each other. **$p < 0.01$ by Student's *t*-test between the responders and non-responders.

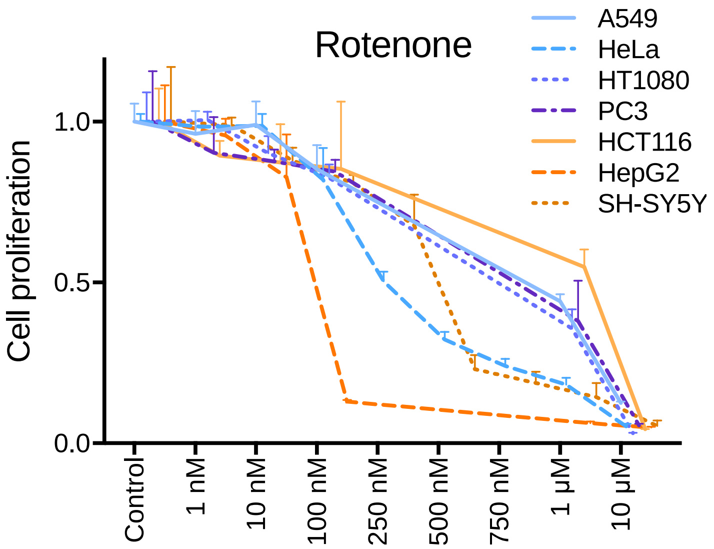

Figure S5. Calibration of proliferation inhibition by rotenone. Relative amount of incorporated BrdU in the seven cell lines under the indicated concentrations of rotenone. The values were normalized for that without rotenone (control). Mean and SD are indicated (*n* = 3 culture dishes).

Table S1. Antibodies for Western blotting.

Molecule	Manufacturer	Host	Cat. No.	Dilution
HSP60	Cell Signaling Technology	Rabbit polyclonal	D307	1:1000
VDAC1	Abcam	Mouse monoclonal	ab14734	1:1000
Phospho-eIf2α (Ser51)	Cell Signaling Technology	Rabbit polyclonal	9721	1:1000
ATF5	Abcam	Rabbit monoclonal	ab184923	1:1000
β-actin	Santa Cruz	Mouse monoclonal	sc-47778	1:1000

Table S2. GEO microarray datasets for gene set enrichment analyses.

Cells	Accession number
A549	2GSM139646
A549	3GSM147365
A549	6GSM335974
A549	9GSM781975_A549_H9N2_0H-1
HeLa	3GSM463942
HeLa	5GSM542389
HeLa	7GSM661462
HeLa	9GSM930228_Cnt_1
HepG2	1GSM1692555_Cont1_HG-U133_Plus_2_
HepG2	5GSM720616_FSHLGC_1
HepG2	6GSM726069_HepG2_APRIL_0h
HepG2	8GSM1366327_Cont1
HT1080	1GSM1443871_1_neg
HT1080	2GSM341404
HT1080	3GSM814750_GR_UPR_HT1080_Cont-2_HG-U133_Plus_2_
HT1080	4GSM887136
HCT116	1GSM1251827_FR_390a21
HCT116	2GSM803635_113404hp133a11
HCT116	4GSM1017469_mRNA_6a_041206
HCT116	5GSM1178133_EA06017_90493_H133+_22
PC3	3GSM377471
PC3	4GSM714354
PC3	5GSM827136
PC3	10GSM1357978_PC3-LPA_1
SH-SY5Y	3GSM231620
SH-SY5Y	5GSM231644
SH-SY5Y	6GSM231654
SH-SY5Y	7GSM231664
